# Protrieve sheath utilization for capturing supra-filter thrombus during a retrieval of thrombosed and embedded IVC filter

**DOI:** 10.1186/s42155-023-00397-4

**Published:** 2023-10-26

**Authors:** Annabella Shewarega, Taylor M. Powell, Douglas Silin

**Affiliations:** 1 Department of Diagnostic and Interventional Radiology and Neuroradiology, University Hospital Essen (AöR), Hufelandstreet 55, 45147 Essen, Germany; 2 Department of Radiology and Biomedical Imaging, Yale School of Medicine, 330 Cedar Street, New Haven, CT 06510 USA

**Keywords:** IVC filter-related thrombosis, Endovascular thrombectomy, Embolizing thrombus, IVC filter removal

## Abstract

**Background:**

Inferior vena cava (IVC) filters, while effective in preventing pulmonary embolism, can increase the risk of IVC thrombosis. IVC filter (IVCF) thrombosis can result from emboli getting trapped within the filter, extension of deep vein thrombosis (DVT), or the device’s inherent thrombogenicity causing in situ thrombosis. This condition can cause noticeable clinical symptoms and complicate the removal of the filter due to the potential for thromboembolism, often resulting in temporary filters remaining unextracted. This case report highlights a novel approach employed to mitigate the risk of thromboembolism during the procedure by capturing mobilized thrombus proximally to the entrapped IVCF.

**Case presentation:**

A 54-year-old woman with a complex medical history including cerebral palsy, Crohn’s disease, and transfusion-dependent iron-deficiency anemia experienced a pulmonary embolism. Due to failed anticoagulation therapy resulting in gastrointestinal bleeding and high transfusion requirements, she underwent placement of an IVCF as a preventive measure against recurrent pulmonary embolism. Three years later, the patient presented with lower extremity swelling and a sudden decline in hemoglobin levels. Diagnostic imaging revealed adherent nonocclusive thrombus within and above the indwelling IVCF. Utilizing the novel Protrieve sheath with the self-expandable Nitinol funnel, successful endovascular removal of the embedded IVCF and adherent thrombus was performed, while mitigating the risk of intraprocedural pulmonary embolism.

**Conclusions:**

The successful intraprocedural trapping and removal of mobilized thrombus from the IVCF removal was achieved using the Protrieve sheath and Nitinol funnel. This approach provides a promising solution to reduce the risk of embolization during the removal of thrombosed IVCFs, potentially outweighing the complications associated with filter removal.

## Background

Vena caval interruption through the percutaneous image-guided insertion of an inferior vena cava (IVC) filter is an important therapeutic option for preventing pulmonary embolism in specific patients with venous thromboembolism (VTE) [1]. The American College of Radiology (ACR) recognizes absolute indications for IVC filters (IVCF) in patients with proven acute VTE who are unable to undergo anticoagulation therapy due to contraindications or recurrent VTE despite therapeutic anticoagulation [2].

Two types of IVCFs are available: permanent and retrievable filters. Permanent filters have been utilized for long-term mechanical prophylaxis against pulmonary embolism when anticoagulation therapy is not feasible. Retrievable filters, introduced in the late 1990s, offer the advantage of removal once the temporary risk of pulmonary embolism or the contraindication to anticoagulation has resolved [3]. Despite their efficacy in reducing the incidence of pulmonary embolism, prolonged placement of IVCFs beyond 30 days has been associated with increased complications [4], including organ penetration, mechanical filter failure, IVC narrowing or blockage, and local thrombosis. Early removal of IVCFs is recommended once the indication for placement has resolved, followed by initiation of anticoagulation therapy [5].

Among retrievable IVCFs, the incidence of IVC thrombosis ranges from 0.6% to 8% [6]. IVC thrombosis can occur due to emboli entrapment within the filter, extension of DVT, or in situ thrombosis caused by the inherent thrombogenicity of the device [7] and can lead to decreased filter patency, impaired venous return from the lower extremities, and ultimately, stasis. The clinical manifestations can vary from asymptomatic cases to the development of post-thrombotic syndrome, characterized by debilitating lower extremity pain, edema, venous claudication, and stasis ulcers [8].

In this article, we present a case of thrombosis related to an embedded IVCF that had been implanted for a period of three years. Retrieval of IVCFs, especially in cases of filter thrombosis, can pose challenges due to the risk of thromboembolism. The Protrieve sheath, a component of the FlowTriever Thrombectomy System (Inari Medical Inc., Irvine, CA), was utilized in this unique case to capture mobilized thrombus during the removal of the thrombosed IVCF.

## Case presentation

**Fig. 1:**
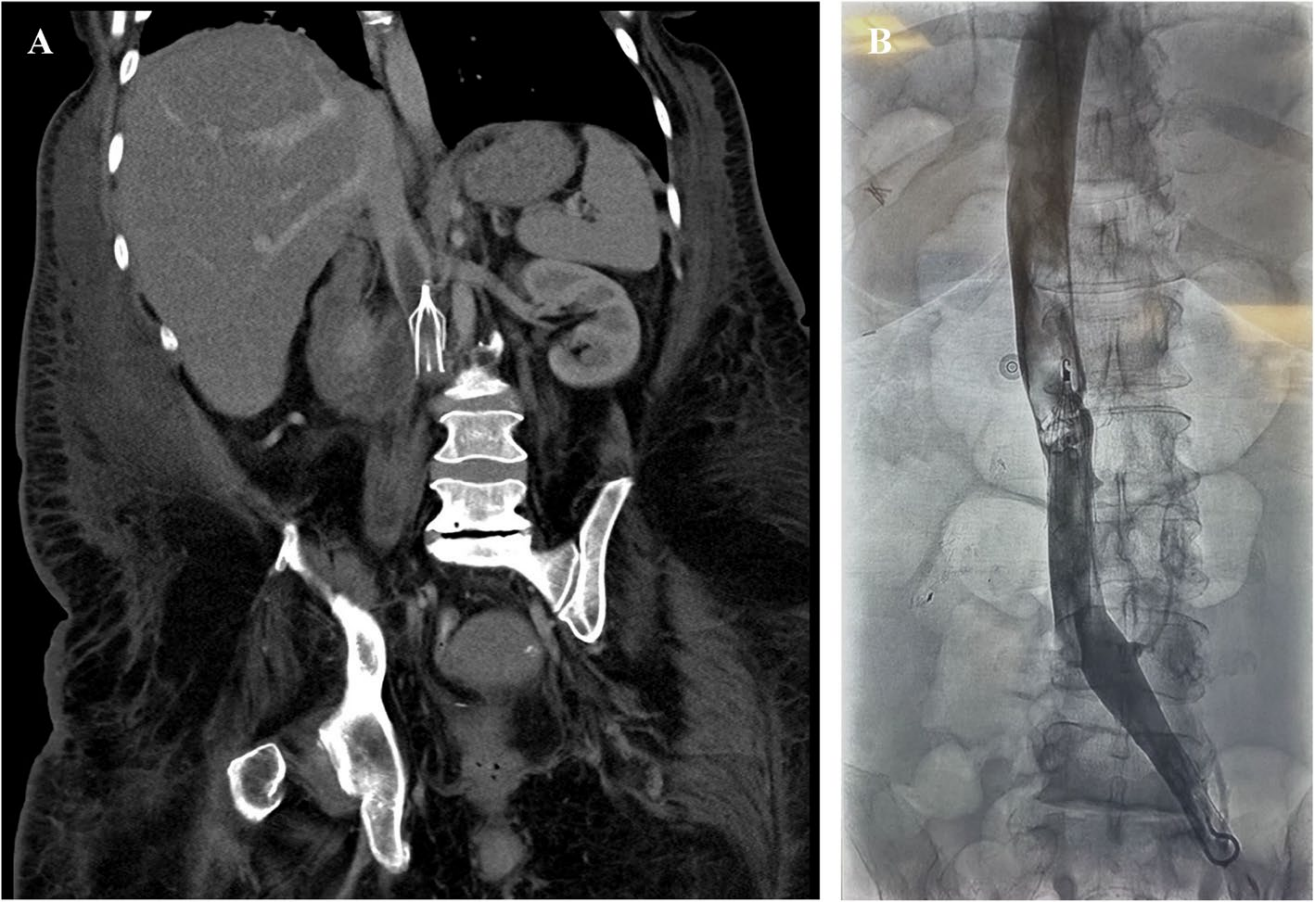
**A** CT abdomen with contrast and **B** fluoroscopy contrast study showing an adherent nonocclusive thrombus within and above an indwelling IVC filter

A 54-year-old woman with a complex medical history including cerebral palsy, Crohn’s disease, and transfusion-dependent iron deficiency anemia experienced bilateral pulmonary embolism. Following thrombectomy, the administration of pharmacologic anticoagulation resulted in gastrointestinal bleeding and a substantial need for blood transfusions. Therefore, a retrievable IVCF was implanted as a preventive measure against recurrent pulmonary embolism. Three years later, the patient experienced generalized weakness and bilateral lower extremity pain. Physical examination revealed noticeable swelling and pain in both lower extremities, particularly upon calf compressions. Overnight, her hemoglobin level dropped from 8.5 g/dL to 7.8 g/dL. A diagnostic CT abdomen study with intravenous contrast revealed an adherent thrombus within and above an indwelling IVCF (Fig. 1A). Endovascular thrombectomy and removal of the IVCF was planned.

The patient was moderately sedated and placed supine. Access was gained to the right internal jugular vein using a 21G micropuncture kit (Cook Medical, Bloomington, IN). A 5F Berenstein catheter (Merit Medical Systems Inc., South Jordan, UT) was advanced through a 6F vascular sheath and used to direct a 0.035-inch hydrophilic-coated guidewire (Glidewire, Cook Medical, Bloomington, IN) beyond the IVCF and into the left common iliac vein. The catheter was exchanged for a 5F Omni Flush catheter (AngioDynamics, Queensbury, NY), through which a digital inferior venocavogram was performed, confirming the adherent nonocclusive thrombus surrounding the indwelling IVCF (Fig. 1B). The Omni Flush catheter was removed over a 0.035-inch super-stiff guidewire (Amplatz wire, Cook Medical, Bloomington, IN), and the access site was sequentially dilated to 24F.

A 24F 50-cm long Inari Protrieve sheath with a 33.5 Nitinol funnel (Inari Medical Inc., Irvine, CA) was advanced over the wire and deployed superior to the IVCF in the hepatic IVC. The Protrieve sheath was placed proximal to the renal veins. The distal end of the sheath was then retracted, allowing the self-expanding nitinol mesh funnel to expand. This funnel exhibited proper apposition to the caval wall to effectively collect any emboli generated during the removal of the IVCF or IVC thrombectomy.

Due to the significant thrombus burden, the FlowTriever System (Inari Medical Inc., Irvine, CA), which consists of the Triever20 (T20) catheter and the FlowTriever catheter, was used to minimize both procedure time and blood loss. The T20 aspiration guide catheter was telescoped through the Protrieve sheath to aspirate the thrombus. The T20’s aspiration mechanism of action relies on the generation of a vacuum within the system by pulling back a 60-mL custom large-bore syringe (Inari Medical Inc., Irvine, CA) that was attached to a side port tubing connector of the sheath. Once established, the vacuum was released by opening the respective port and extracting possible thrombus via powerful suction into the syringe. Each 60-mL aspiration was followed by filtration of the blood with the FlowSaver Blood Return system with a 40-μm filter (Inari Medical Inc., Irvine, CA) and returned to the patient via the Protrieve sheath. Despite attempts to aspirate the thrombus connected to the filter, the filtration of the aspirated blood did not reveal any clots. As a result, the next approach focused on removing the thrombosed filter.

**Fig. 2:**
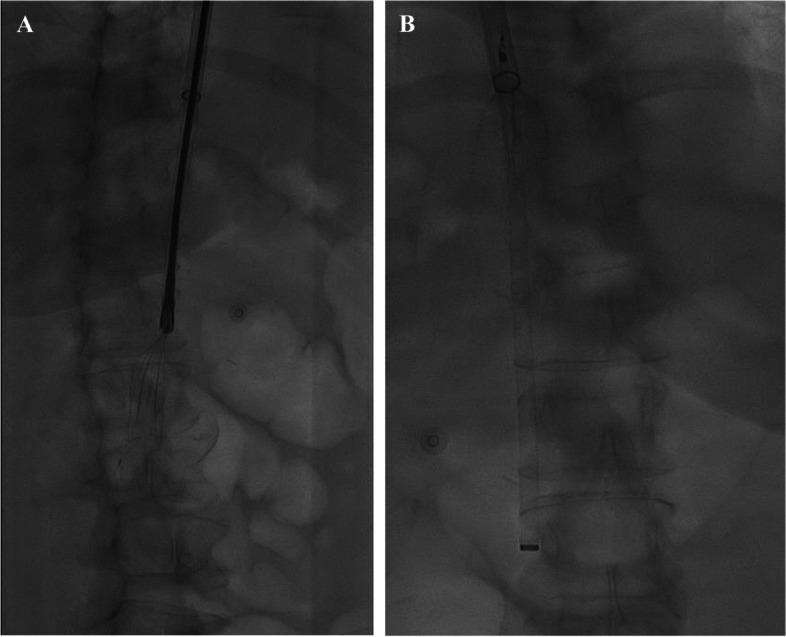
**A** An endobronchial forceps grabbing and partially pulling the indwelling IVC filter into a 14 French sheath. A Protrieve 24 F sheath with a 33.5 mm Nitinol funnel is deployed here but poorly visualized. **B** A small cloverleaf snare snaring the indwelling IVC filter and retrieving the filter through the sheath. The Protrieve 24 F sheath with a 33.5 mm Nitinol funnel is deployed here and well visualized

**Fig. 3:**
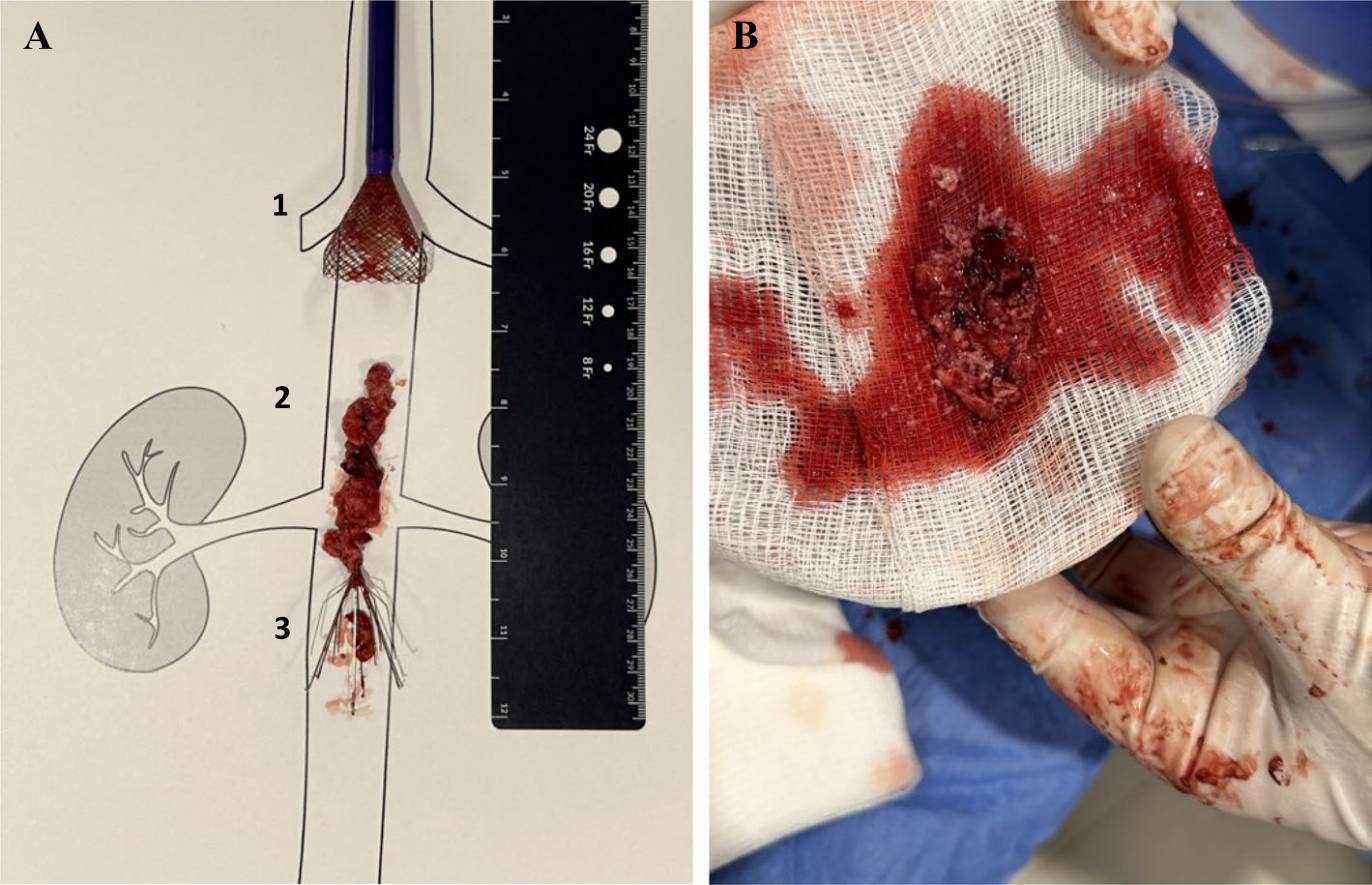
**A** A 24F Inari Protrieve sheath (Inari Medical Inc., Irvine, CA) used for thrombus aspiration. The 33.5 Nitinol funnel (1), retrieved clot (2), and IVC filter (3) are depicted ex-vivo on an anatomical drawing of the abdominal IVC. **B** A moderate burden of chronic appearing thrombus

The attempts to remove the IVCF using a 20-mm gooseneck snare (Merit Medical Systems Inc., South Jordan, UT), advanced through a 9F sheath (Cook Medical, Bloomington, IN) within the 24F Protrieve sheath, proved to be unsuccessful. This revealed an embedded IVCF within the intimal layer of the IVC. Subsequently, the snare and 9F sheath were replaced with a 14F GORE® DrySeal sheath (Gore Medical, Newark, DE), which was positioned coaxially through the 24F sheath. An endobronchial forceps (Lymol Medical, Woburn, MA) was employed successfully to mobilize and grasp the filter (Fig. 2). While attempting to extract the filter into the sheath, the filter detached from the forceps, leading to an incomplete removal. A 3-mm cloverleaf snare (Merit Medical Systems Inc., South Jordan, UT) was advanced through the sheath to retrieve and successfully extract the remaining filter fragments (Fig. 3). Subsequently, both the filter and the 14F sheath were removed together.

**Fig. 4:**
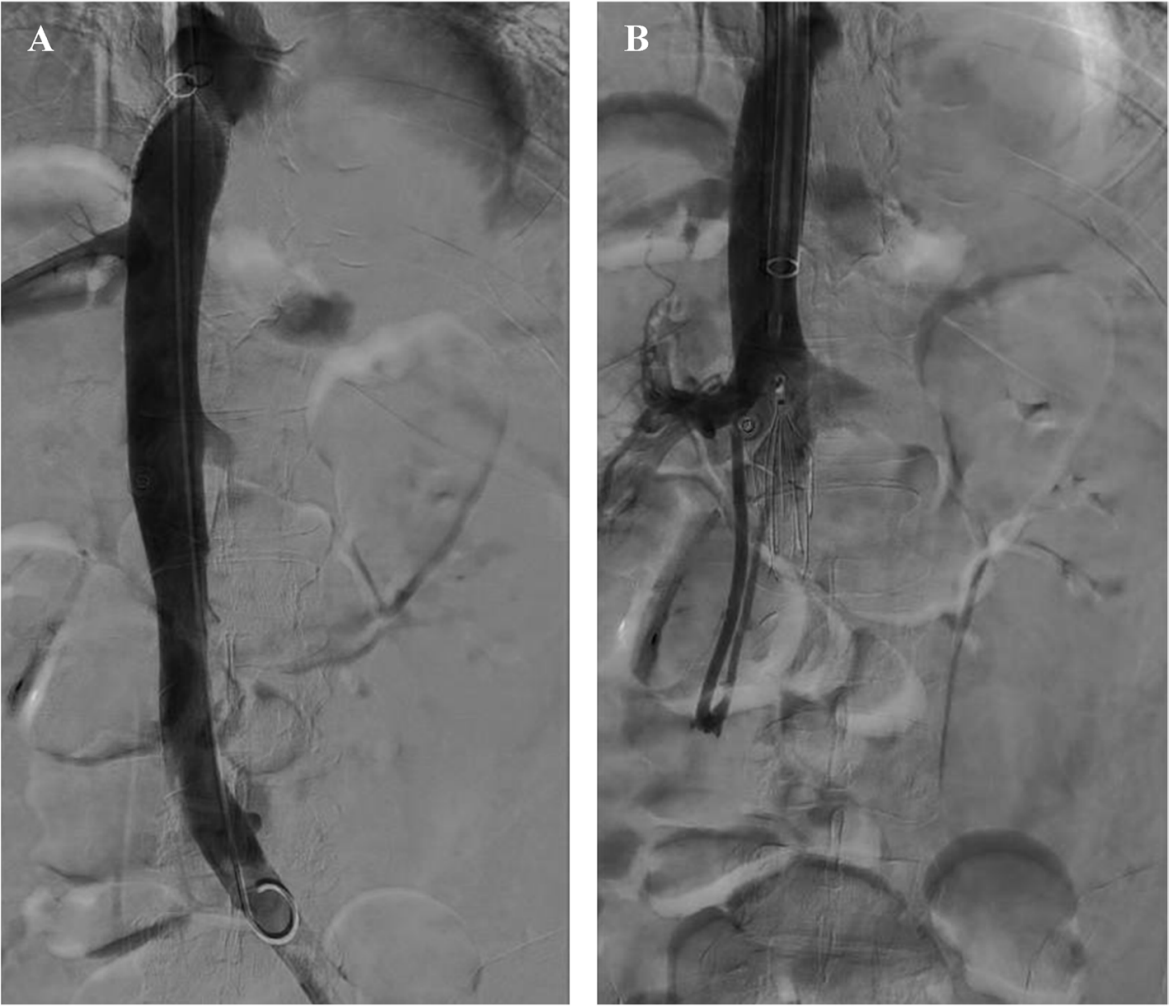
**A** Cavography post-retrieval of the IVC filter and thrombus, and **B** after placement of a new Bard Denali IVC filter (BD, Franklin Lakes, New Jersey)

Thrombus freed from the filter collected within the Protrieve funnel. Aspiration with the T20 aspiration guide catheter through the Protrieve sheath removed the trapped thrombus (Fig. 4). Venocavogram was then performed from the lower IVC, demonstrating no residual thrombus or venous extravasation following filter and thrombus retrieval (Fig. 4A).

To address the ongoing need for an IVCF, the decision was made to proceed with the placement of a Bard Denali IVCF (BD, Franklin Lakes, NJ). To accommodate the length requirements, the Protrieve sheath was replaced with a standard 24F Inari sheath (Inari Medical Inc., Irvine, CA). Subsequently, the delivery sheath of the Bard Denali IVCF was positioned over a wire and inserted into the IVC. The Bard Denali filter was then advanced through the delivery sheath and deployed in the infrarenal IVC. The delivery sheath was guided to the level of the filter, and a brief post-placement inferior venacavogram was performed to confirm the accurate positioning of the filter (Fig. 4B).

Following the removal of wires and sheaths, hemostasis was established at the right neck by employing a 0 Prolene pursestring suture and applying compression. A sterile dressing was applied. The patient tolerated the procedure well, experiencing no bleeding events and showing symptom improvement, and was discharged on post-procedure day three on apixaban. The scheduled one-month follow-up appointment to address IVCF removal was missed. Three months later, the patient’s primary care physician reported no recurrence of symptoms.

## Discussion

This case report highlights the successful removal of a chronically implanted IVCF and an adherent supra-filter thrombus using the novel Protrieve sheath with the Nitinol funnel. Complications associated with retrieval are significantly increased with longer dwell times, increased tilt angles, hook embedment, and filter thrombosis [9]. The retrieval of thrombosed IVCFs can be particularly challenging due to the risk of thromboembolism which often leads to temporary filters remaining unretrieved. In this case, the use of the 24F Inari Protrieve sheath with the self-expandable 33.5 Nitinol funnel proved to be effective in collecting any emboli generated during the removal of the IVCF and IVC thrombectomy, thus mitigating intraprocedural pulmonary embolism.

Pharmacologic anticoagulation, when clinically feasible, remains the mainstay of treatment for IVC thrombosis. Catheter-directed therapy (CDT) enables a localized delivery of thrombolytic agents directly into the thrombus, which may facilitate restoration of venous patency without the increased risk of bleeding complications. Although pharmacologic anticoagulation therapy or CDT have been used, the efficacy of these treatments on the regression or resolution of IVCF-associated thrombus or the occurrence of PE in patients with IVCF thrombosis is still uncertain [10]. Many patients treated for IVCF thrombosis, particularly those with chronic symptoms, may show significant residual clot after thrombolysis. In patients with chronic IVC thrombosis, endovascular modalities, specifically mechanical thrombectomy, balloon angioplasty, and stent placement, may be the best option to restore venous patency. Vedantham et al. assessed and reported these techniques to be both safe and effective in the short term, with a success rate comparable to that reported for iliofemoral DVT therapy [11].

Thromboembolism or the embolization of filter fragments is a persistent concern during endovascular filter retrieval procedures. These risks may lead to doubts about the appropriateness of removal. Nevertheless, the availability of the Protrieve device has the potential to shift the balance in favor of extraction. In this patient’s case, she had encountered complications associated with the filter and was at continued risk of experiencing more. The use of the funnel-shaped sheath reduced the associated risks during removal. Consequently, in this specific scenario, filter retrieval with embolic protection was deemed necessary.

## Conclusion

By employing a mechanical thrombectomy sheath with a self-expandable 33.5 Nitinol funnel, percutaneous removal of a thrombosed IVCF was successfully accomplished, obviating the need for an open procedure. This innovative approach not only addresses the need for removing thrombosed IVCFs, but also mitigates the risk of complications arising from embolizing thrombosis. By effectively capturing and removing thrombus, this approach offers a comprehensive solution that promotes patient safety and successful management of IVCF-related thrombosis.

## Data Availability

Data sharing is not applicable to this article as no datasets were generated or analysed during the current study.

## Abbreviations

ACR: American College of Radiology
DVT: Deep vein thrombosis
F: French
IVC: Inferior vena cava
IVCF: Inferior vena cava filter
VTE: Venous thromboembolism

## References

[1] DeYoung E, Minocha J (2016). Inferior Vena Cava filters: guidelines, best practice, and expanding indications. Semin Intervent Radiol.

[2] Minocha J, Smith AM, Kapoor BS (2019). ACR appropriateness criteria® Radiologic management of venous thromboembolism-inferior vena cava filters. J Am Coll Radiol.

[3] Marron RM, Rali P, Hountras P, Bull TM (2020). Inferior vena cava filters: past, present, and future. Chest.

[4] Angel LF, Tapson V, Galgon RE, Restrepo MI, Kaufman J (2011). Systematic review of the use of retrievable inferior vena cava filters. J Vasc Interv Radiol.

[5] Kelkar AH, Rajasekhar A (2020). Inferior vena cava filters: a framework for evidence-based use. Hematology.

[6] Shah NG, Wible BC, Paulisin JA (2021). Management of inferior vena cava thrombosis with the FlowTriever and ClotTriever systems. J Vasc Surg Venous Lymphat Disord.

[7] Okutucu S, Ates I, Marmagkiolis K, Kose G, Iliescu C, Cilingiroglu M (2020). Successful WATCHMAN device implantation in a patient with IVC filter thrombosis and iliac vein occlusions. Cardiovasc Revascularization Med.

[8] Kahn SR (2016). The post-thrombotic syndrome. Hematol Am Soc Hematol Educ Prog.

[9] Andreoli JM, Thornburg BG, Hickey RM (2016). Inferior vena cava filter–related thrombus/deep vein thrombosis: data and management. Semin Intervent Radiol.

[10] Alkhouli M, Zack CJ, Zhao H, Shafi I, Bashir R (2015). Comparative outcomes of catheter-directed thrombolysis plus anticoagulation versus anticoagulation alone in the treatment of inferior vena caval thrombosis. Circulation: Cardiovasc Intervent.

[11] Vedantham S, Vesely TM, Parti N (2003). Endovascular recanalization of the thrombosed filter-bearing inferior vena cava. J Vasc Interv Radiol.

